# Lipid Metabolism and Improvement in Oilseed Crops: Recent Advances in Multi-Omics Studies

**DOI:** 10.3390/metabo13121170

**Published:** 2023-11-23

**Authors:** Mengjia Bu, Wei Fan, Ruonan Li, Bing He, Peng Cui

**Affiliations:** 1 Agricultural Genomics Institute at Shenzhen, Chinese Academy of Agricultural Sciences, Shenzhen 518124, China; 2 College of Informatics, Huazhong Agricultural University, Wuhan 430070, China

**Keywords:** oilseed crops, lipid metabolism, seed oil content, multi-omics

## Abstract

Oilseed crops are rich in plant lipids that not only provide essential fatty acids for the human diet but also play important roles as major sources of biofuels and indispensable raw materials for the chemical industry. The regulation of lipid metabolism genes is a major factor affecting oil production. In this review, we systematically summarize the metabolic pathways related to lipid production and storage in plants and highlight key research advances in characterizing the genes and regulatory factors influencing lipid anabolic metabolism. In addition, we integrate the latest results from multi-omics studies on lipid metabolism to provide a reference to better understand the molecular mechanisms underlying oil anabolism in oilseed crops.

## 1. Introduction

Most of the lipids consumed worldwide are produced by plants [1]. Lipids—the most energy-dense storage materials in plants—are usually stored in seeds and provide calories and essential fatty acids needed for the human diet. In addition, many fractions from seed meal (ground whole seeds), such as the seed fraction left after oil removal, are used as protein-rich animal feeds [2]. Furthermore, vegetable oils can replace petroleum, including as biofuels and as feedstock (raw materials) for the chemical industry [3,4]. Demand for high-quality seed oils continues to grow, and global oilseed production is expected to reach a record 638.4 million tons in 2022/23, according to the Food and Agriculture Organization’s (FAO) biannual Food Outlook (FAO, 2022). Increasing yields and improving quality through better farming techniques and plant breeding can help meet this growing demand for oil crops [5]. Increasing the oil content in oilseed crops through breeding is an appealing way to improve oil yield, and this approach requires a thorough understanding of the gene networks involved in oil biosynthesis during seed development. In this review, we detail the latest knowledge about plant lipids, including their function, their classification in plants, and the general mechanisms of accumulation and storage in plants. After that, we discuss the research progress of key genes of lipid metabolism in plants, including new genes and transcription factors involved in lipid metabolism and regulation, and more importantly, the research progress of omics in oil crops. This work provides information for improving the oil content in seeds of oil species at the molecular level.

## 2. Current Knowledge on Plant Lipids

### 2.1. Function of Lipids

Lipids play many roles in plant cells. For example, polar glycerides are the major components of cell membranes, where they act as protective barriers against external damage and initiate signaling [6].

Lipid composition varies widely among different species and tissues [7]. For example, plants accumulate large amounts of lipids in their seeds or fruits to provide the energy needed for germinating seeds, maintaining moisture, and preventing cold cracking and frostbite in the seeds. Lipids play essential roles in cell signal transduction between flowers, leaves, stems, and other tissues [8] and are deposited as waxes on the plant surface to decrease non-stomatal plant water loss or ultraviolet (UV) damage, thus increasing plant tolerance to abiotic stress [9]. In many plants, lipids account for 5% to 10% of the total dry weight, with even higher proportions found in seeds and other storage organs [10].

### 2.2. Classification of Plant Lipids

**Figure 1:**
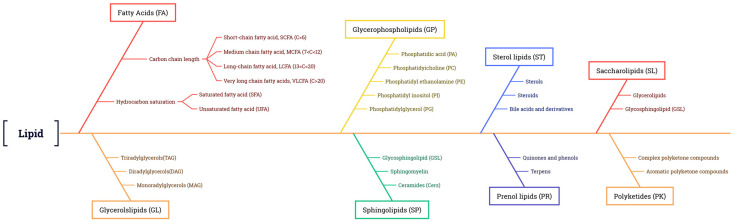
Classification of lipids.

Lipids are widely distributed in all plant tissues and can be divided into eight types according to their chemical composition: FAs, glycerol lipids, glycerophospholipids, sphingolipids, sterol lipids, allyl alcohol lipids, glycolipids, and polyketides [11,12] (Figure 1). FAs are the building blocks of many of the more complex lipids. About 40 FAs serve as the major components of natural lipids, among over 10,000 known fatty acyl molecules (https://www.lipidmaps.org) accessed on 22 September 2018 [11,13]. When supplied with sufficient oxygen, FAs can be oxidized and broken down into CO_2_ and H_2_O, releasing large amounts of energy, thus making them a major source of energy. FAs can be further divided into saturated fatty acids (SFAs) and unsaturated fatty acids (UFAs) based on the absence or presence of double bonds. In most plants, the major SFAs are palmitic acid (16:0, 16 is the number of carbon atoms, 0 represents the number of double bonds) and stearic acid (18:0), and the major UFAs are monounsaturated FAs (MUFAs), such as oleic acid (18:1), and polyunsaturated FAs (PUFAs), such as linoleic acid (LA, 18:2) and α-linolenic acid (α-ALA, 18:3) [14]. These five FAs account for approximately 90% of the lipids in commercial vegetable oils on the market [15].

MUFAs are straight-chain aliphatic compounds with a single carbon–carbon double bond at some positions in the chain; over 50 of such molecular structures have been documented in plants (https://plantfadb.org) accessed on 19 October 2023 [13]. The location and geometry of the unsaturated double carbon–carbon bond gives membrane, storage, and surface lipids their specific properties [16]. The molecular structure of PUFAs contains two or more double bonds (unsaturated bonds). Many PUFAs are essential to the human diet and are beneficial to human health [17]. In particular, ω-3 PUFAs contribute to the prevention of cardiovascular and inflammatory diseases [18]. Three PUFAs essential for the human diet are LA, α-ALA, and arachidonic acid, and they are obtained from plant sources [19].

In addition to these common FAs, plants also produce more than 450 types of unusual FAs with different chain lengths, numbers and positions of double bonds, and unique side groups [13]. These FAs perform various specific functions based on their unique chemical properties. Specifically, fatty acid hydroxylase 12 (RcFAH12) from castor bean (*Ricinus communis*) converts phosphatidylcholine (PC)-bound 18:1 to 18:1-OH to produce hydroxy fatty acids that can be used as plastics and lubricants [20].

**Figure 2:**
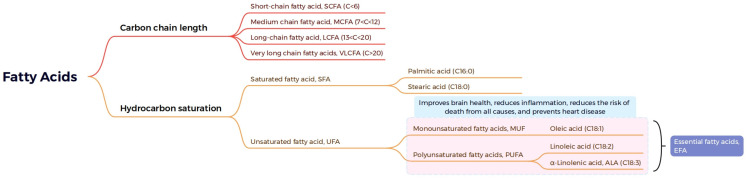
Detailed classification of fatty acids, the major components of plant lipids.

FAs can also be divided into different groups according to their carbon chain length: short-chain FAs (C < 6), medium-chain FAs (C = 7–12), long-chain FAs (C = 13–20), and very-long-chain FAs (VLCFAs; C > 20) [21]. Most FAs have even-numbered chain lengths, and only a few have odd-numbered chain lengths, such as C15 and C17 [22]. VLCFA biosynthesis begins with the elongation of saturated C16 and monounsaturated C18 FAs, which are produced in the plastids and exported to the cytosol, where they are activate as acyl-CoAs by long-chain acyl-CoA synthetases (LACSs) [23]. VLCFAs are found exclusively in several membrane lipids and are essential for membrane homeostasis. In epidermal cells, VLCFAs are the precursors of plant cuticle waxes and are of primary importance for many plant interactions with their surrounding environment [24]. VLCFAs are also a major component of the root subcortical barrier, which is fundamental for nutrient homeostasis and plant adaptation to adverse conditions [25] (Figure 2).

### 2.3. Lipid Accumulation and Storage in Plants

#### 2.3.1. De Novo Biosynthesis of FAs

In oilseed crops, the first step in FA biosynthesis is the conversion of acetyl-CoA produced via sucrose glycolysis to malonyl-CoA by acetyl-CoA carboxylase (ACCase). ACCase is a type I biotin-containing enzyme and there are two main forms of ACCase in plants: Heteromeric ACCaseI and Homomeric ACCaseII. ACCase is composed of four subunits: biotin carboxylase (BC), biotin carboxyl carrier protein (BCCP), α-carboxyltransferase (α-CT), and β-carboxyltransferase (β-CT). It has three functional domains, namely the BC functional domain, the BCCP functional domain, and the CT functional domain, respectively [26,27]. After its biosynthesis, malonyl-CoA is transferred to the ACP component of the fatty acid synthase (FAS) complex by malonyl-CoA:ACP malonyltransacylase (MCMT). In plants, FAS is a multi-component type-II enzyme located in the plastids, consisting of 3-β-ketoacyl-ACP synthase III (KASIII), β-ketoacyl-ACP synthase (KASI), ketoacyl-ACP reductase (KAR), hydroxyacyl-ACP reductase (HAD), and enoyl-ACP reductase (ENR). FAS uses acetyl-CoA as the starting unit for a condensation reaction. Each elongation cycle is supplied with a two-carbon unit by malonyl-ACP to produce 16:0-ACP and 18:0-ACP after seven or eight cycles, respectively, at which point 18:0-ACP passes through Δ9 stearoyl-ACP desaturase to form 18:1-ACP, making phosphatidic acid (PA) (16:0) and oleic acid (18:1) the major products of FA biosynthesis in most plant plastids [28]. After biosynthesis, ACP is removed by fatty acyl-ACP thioesterase (FATB/FATA) to produce free FAs (non-esterified FAs, NEFAs) that are then transported through the plastid envelope and converted by LACS to 16:0 and 18:1 CoA, which are used for TAG biosynthesis in the endoplasmic reticulum (ER) [15].

#### 2.3.2. TAG Biosynthesis

In most oilseed crops, such as soybean (*Glycine max*) and rapeseed (*Brassica napus*), TAGs are stored in seeds as an energy source for germination and aid in seed dispersal [29]. TAG consists of a glycerol backbone and three FA molecules chemically linked by ester bonds, providing a carbon skeleton and energy source. TAG biosynthesis and accumulation occur through a complex network of reactions taking place in the plastid, cytoplasm, and ER. Depending on the plant species, TAG can accumulate in different organs, mainly in embryonic tissues (rapeseed) or endosperm tissues (castor bean). In oilseeds that store oil in the embryo, the main storage tissue is the cotyledons, but substantial seed oil can also accumulate in the hypocotyl, radicle, and surrounding endosperm/aleurone layers [30]. After acyl-CoA is transported from the plastid to the ER, the most common pathway for TAG biosynthesis is the acyl-CoA-dependent Kennedy pathway [31]. In this pathway, acyl-CoA is incorporated into glycerol-3-phosphate (G3P) by acyl-CoA:glycerol-3-phosphate acyltransferase (GPAT) and lysophosphatidic acid acyltransferase (LPAT) at the sn-1 and sn-2 positions of G3P, respectively, to form PA. Subsequently, phosphatidic acid phosphatase removes one phosphate group from PA, and the resulting DAG is converted by acyl-CoA:diacylglycerol acyltransferase (DGAT) via acylation at the sn-3 position. TAG biosynthesis is not a simple linear pathway because TAG composition depends on the flux of PC prior to the incorporation of FAs into TAG [32]. As an important phospholipid, PC is involved in the assembly and regulation of cell membrane structure. Its alterations can affect the lipid balance within the cell, thereby influencing the composition of TAGs.

#### 2.3.3. Formation of Lipid Droplets

Cytoplasmic lipid droplets (LDs) are organelles that store non-polar lipids such as TAGs and sterol esters [33]. In mature seeds, LDs are distributed in the central region of storage cells and are mostly oval or irregular in shape [34]. The primary LD structure consists of a phospholipid monolayer coated with various proteins. The current general model of LD biogenesis is that non-poplar lipids such as TAGs are first produced by membrane-associated enzymes in the ER and then accumulate in the form of a lens between lobes of the ER membrane, culminating with LD formation on the cytoplasmic side of the ER membrane [35]. Two important proteins have recently been shown to be involved in LD formation: SEIPIN [36,37] and lipid-droplet-associated protein in Arabidopsis (*Arabidopsis thaliana*) [38].

#### 2.3.4. Inhibition of Lipid Degradation

**Figure 3:**
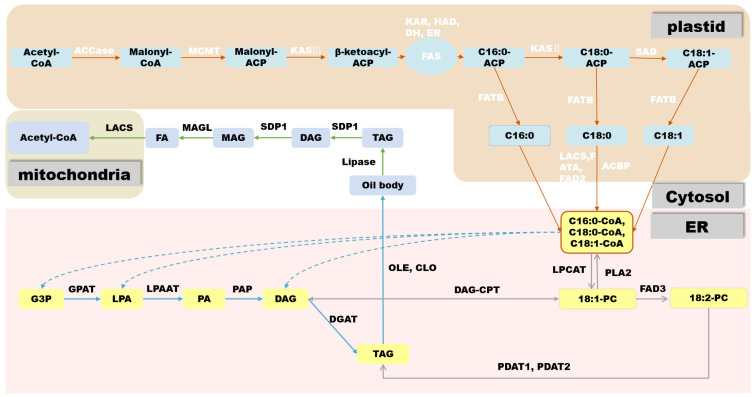
General overview of fatty acid biosynthesis in oilseed crops. ACCase: Acetyl-CoA Carboxylase; MCMT: Monoacylglycerol acyltransferase; KASIII: β-ketoacyl-ACP; KAR: β-ketoacyl-ACP reductase; HAD: β-hydroxyacyl-ACP dehydrogenase; DH: enoyl-ACP dehydratase; ER: enoyl-ACP reductase; KASII: 3-ketoacyl-ACP synthase II; SAD: stearoyl-ACP desaturase; FATB: Fatty Acid Thioesterase B; LACS: Long-chain acyl-CoA synthetase; FATA: Fatty Acid Thioesterase A; FAD2: Fatty acid desaturase 2; ACBP: Acyl-CoA binding protein; LPCAT: Lysophosphatidylcholine acyltransferase; PLA2: Phospholipase A2; FAD3: Delta-15 desaturase; DAG-CPT: Diacylglycerol cholinephosphotransferase; PDAT: Phospholipid: Diacylglycerol Acyltransferase; OLE: Oleate desaturase; CLO: Linoleate desaturase; SDP1: sn-glycerol-3-phosphate dehydrogenase; MAGL: Monoacylglycerol lipase; DGAT: Diacylglycerol acyltransferase; PAP: Phosphatidate phosphatase; LPAAT: Lysophosphatidic acid acyltransferase; GPAT: Glycerol-3-phosphate acyltransferase.

Oil accumulation in seeds involves a dynamic balancing act between lipid biosynthesis and degradation. Due to lipid degradation, the oil content of rapeseed decreases by about 10% during the final stages of seed development, resulting in an estimated loss of approximately 20 million tons of vegetable oil per year [39]. The two types of enzymes responsible for lipid degradation are lipases and lipoxygenases [40]. Lipid degradation via lipases produces glycerol and NEFAs [41]. FAs are transported to the mitochondria and glyoxylate cycle bodies for β-oxidation and then the glyoxylate cycle. β-Oxidation allows NEFAs to be converted to acetyl-CoA, and complete oxidation of acetyl-CoA occurs via the tricarboxylic acid cycle [42,43] (Figure 3).

## 3. Progress in the Identification of Key Genes behind Lipid Metabolism in Plants

Over the past 70 years, much progress has been made in identifying the corresponding enzymes and genes required for FA biosynthesis, with Arabidopsis being the most highly studied plant system [28]. Indeed, over 600 genes related to lipid metabolism have been successfully annotated in Arabidopsis [44]. The rapid development of high-throughput sequencing technologies now makes it possible to conduct similar comprehensive studies on lipid metabolism in a variety of oilseed species.

### 3.1. Identification and Functional Characterization of Key Genes

As mentioned above, FATB is a key enzyme in FA biosynthesis that catalyzes the removal of ACP from 16:0-ACP and 18:1-ACP to generate NEFAs. The knockdown of *GmFATB1* expression decreases UFA content in soybean seeds [45]. Similarly, the expression of *BnFATB* in rapeseed appears to be highly and positively correlated with seed oil content (SOC), as evidenced by an analysis integrating quantitative trait loci (QTLs) from nine different populations [46].

LPAT is one of the key enzymes in the Kennedy pathway that converts acyl-CoA to PA. The heterologous expression of a yeast (*Saccharomyces cerevisiae*) sn-2 acyltransferase gene in rapeseed resulted in increased FA content by 8–22% [47]. Cloning and spatiotemporal expression of *AhLPAT2* from peanut (*Arachis hypogaea*) showed that increased expression of this gene was closely related to seed oil content; moreover, heterologous expression of *AhLPAT2* in Arabidopsis from a seed-specific promoter significantly increased SOC [48]. 

DGAT is a key enzyme that acylates DAG at the sn-3 position. Seed-specific overexpression of Arabidopsis *DGAT* in wild-type plants resulted in an increase in SOC and seed weight from 29% to 35%, along with an increase in the average thousand-seed weight from 19 mg in non-transgenic plants to 23 mg in the overexpression lines [49], which was consistent with the results of heterologous expression of *DGAT1* from sesame (*Sesamum indicum*) in Arabidopsis [50]. An investigation of the expression of the four copies of *BnDGAT1* in developing seeds from 34 inbred rapeseed lines revealed that each copy exhibits different expression patterns in different germplasm resources; notably, higher expression of *BnDGAT1* genes was positively correlated with SOC [51].

GDSL esterase is a type of hydrolase that is widely involved in various physiological activities in plants and is important in oil metabolism. The name of the enzyme derives from its conserved domain (GDSL), where G, D, and S represent glycine (Gly), aspartate (Asp), and serine (Ser), respectively [52]. Arabidopsis has five GDSL-like lipase genes (also called *SEED FATTY ACID REDUCER* [*SFAR*] genes) that decrease SOC by acting downstream of the gibberellin signaling (GA) pathway since GA may affect the seed FA content via SFARs [53]. The overexpression or knockout of each *SFAR* gene significantly lowered or increased SOC, respectively, and altered FA composition. The heterologous expression of a *GDSL* gene from oil palm (*Elaeis guineensis*) in Arabidopsis resulted in a 9.5% increase in total FA content compared to the wild type. Further analysis of the FA composition revealed that stearic acid (18:0) increased in the seeds of the transgenic lines. The overexpression of *EgGDSL* should lead to a decrease in SOC in the transgenic lines compared to the wild type [54]. 

In addition to these important pathway enzymes and their encoding genes directly involved in de novo FA biosynthesis and TAG assembly, recent studies have identified several genes annotated as participating in carbon source provisioning and photosynthetic pathways that may also be involved in regulating SOC [55]. For instance, trehalose-6-phosphate (T6P), a metabolic precursor of sucrose and a key signaling molecule for plants to respond to carbon availability and regulate growth and development, also regulates FA biosynthesis by inhibiting sucrose non-fermenting 1-related kinase 1 (SnRK1). Indeed, incubation of rapeseed suspension cells in T6P-containing medium or heterologous expression of *T6P synthase* from *Escherichia coli* in *Nicotiana benthamiana* significantly increased FA biosynthesis rates [56]. CALCINEURIN B-LIKE PROTEIN-INTERACTING PROTEIN KINASEs (CIPKs) are a family of energy-signaling protein kinases in plants. Relative to non-transgenic plants, the overexpression of *BnCIPK9* during seed development in transgenic rapeseed lowered oil biosynthesis [16]. The SOC of *cipk9* mutants was higher than that of wild-type Arabidopsis plants and was rescued by the introduction of a complementation construct consisting of the *AtCIPK9* promoter and *AtCIPK9* coding region, indicating that AtCIPK9 negatively regulates lipid accumulation and significantly affects the early establishment of Arabidopsis seedlings.

### 3.2. Transcription Factors Involved in Regulation

Looking for transcription factors (TFs) that regulate the expression of multiple genes is also an effective method to increase SOC [57]. Currently, well-studied TFs in plants mainly include WRINKLED1 (WRI1), LEAFY COTYLEDON1 (LEC1), LEC2, LEC1-LIKE (LIL), FUSCA3 (FUS3), and ABSCISIC ACID INSENSITIVE3 (ABI3).

WRI1 is an APETALA2 (AP2)/ETHYLENE-RESPONSIVE ELEMENT BINDING PROTEIN (EREBP) that regulates genes involved in glycolysis and sucrose entry into TAG [58]. WRI1 was discovered in Arabidopsis in 1998 as a mutant affecting seed storage accumulation. Compared with the wild type, the mutant was unable to convert glucose and sucrose into precursors for fatty acid synthesis during seed development and reduced the activity of several glycolytic enzymes such as hexokinase and phosphofructokinase, resulting in an 80% reduction in SOC [59]. The oil content of *N. benthamiana* leaves expressing *WRI1* from castor bean showed a 4.3–4.8 times increase compared to the control group. The Arabidopsis *wri1* loss-of-function mutant was almost completely rescued by the strong expression of castor bean *WRI1* from a seed-specific promoter, resulting in a total FA content close to that of non-transgenic seeds [60]. LEC1 is a member of the nuclear factor-YB (NF-YB) family of TFs, and the individual overexpression of Arabidopsis *LEC1* or *L1L*, or the overexpression of their homologous genes *BnLEC1* or *BnL1L* from rapeseed, resulted in significantly increased FA levels in transgenic Arabidopsis [61,62]. LEC2 is a plant-specific B3-type TF; high heterologous expression of *GmLEC2* in the Arabidopsis *lec2* mutant largely rescued the defect in TAG accumulation, increasing TAG and long-chain FA contents by 34% and 4%, respectively [63].

FUS3, another member of the plant-specific B3 domain family, plays an important role in recognizing and binding to the RY element CATGCA, which is found in the promoters of many genes. *fus3* mutants in rapeseed showed a lower total SOC than the wild type [64]. Inducible expression of Arabidopsis *FUS3* increased the oil content of Arabidopsis seedlings to 6% of dry weight, which was more than 50-fold higher than that of non-transgenic seeds. Similarly, the inducible expression of *FUS3* in *Nicotiana tabacum* L. cv Bright Yellow2 (BY2) cells increased TAG accumulation, and the co-expression of *FUS3* and *DGAT1* further increased the TAG levels to 4% of the dry weight. A microarray analysis of wild-type and *FUS3*-overexpressing Arabidopsis seedlings showed that *FUS3* overexpression was associated with the increased expression of many genes involved in TAG biosynthesis rather than genes involved in FA biosynthesis or other lipid pathways [65]. 

ABI3 is also a member of the B3 domain TF family. The heterologous expression of *GmABI3* from soybean in Arabidopsis significantly increased TAG content and altered the FA composition of seeds. In addition, *GmABI3* expression successfully complemented the phenotype and oil content of Arabidopsis *abi3* mutant seeds [66]. In another study, tobacco leaf protoplasts were prepared for transfection with effector constructs overexpressing *WRI1* and/or *DGAT1* alone or together with one of the master regulators *ABI3*, *FUS3*, *LEC1*, or *LEC2.* The co-expression of *FUS3* and *ABI3* with *WRI1* and *DGAT1* significantly increased the content of total non-polar lipids of the protoplasts. Notably, the expression of *ABI3* overexpression alone resulted in the highest accumulation of total non-polar lipid in protoplasts. Furthermore, in contrast to *LEC2*, the co-expression of *ABI3* further increased the lipid content of protoplasts transiently expressing *WRI1* and *DGAT1* [67]. In addition to the regulatory roles of single TFs, the cross-regulatory effects of these TFs have also been observed. For example, ABI3, together with LEC1, LEC2, and FUS3, may form the so-called LAFL (LEC1, ABI3, FUS3, and LEC2) regulatory network required for storage material accumulation during seed development [68,69].

MYB TFs comprise one of the largest gene families in plants and are associated with the regulation of plant growth and development, metabolism, morphology, and cellular patterning [70]. MYB92 can directly bind to the promoter of and activate the transcription of *Biotin carboxyl carrier protein 2* (*BCCP2*), which encodes a component of the FA biosynthetic pathway. The overexpression of Arabidopsis *MYB92* in *N. benthamiana* induced the expression of FA biosynthesis genes, resulting in the accumulation of various types of lipids [71]. DECREASE WAX BIOSYNTHESIS 2 (DEWAX2) is a member of the AP2-EREBP TF family in Arabidopsis and negatively regulates epidermal wax deposition by directly binding to the promoters of *LACS1*, *LACS2*, *KCSII*, and *ECERIFERUM1* (*CER1*) to inhibit their expression [9]. Another important TF family is the WRKY family, which plays an important role in plant stress regulatory networks involved in responses to biotic and abiotic stress. The loss of WRKY6 function in Arabidopsis resulted in a significant increase in seed size along with an increase in FA content and composition. Ultrastructural analysis revealed that the *wrky6* mutant also accumulated more LDs in the cells of mature seeds, which altered the expression of several genes related to photosynthesis and FA biosynthesis at 10 or 16 days after pollination [72]. 

*Growth-regulating factor 2a* (*BnRGF2a*) was identified in rapeseed based on its differential expression between two rapeseed lines with differing seed oil production. Arabidopsis lines overexpressing *BnGRF2* showed improved seed quality and oil content. In addition, transcriptome analysis revealed that some genes related to cell proliferation, photosynthesis, and oil biosynthesis were upregulated in these overexpression lines, suggesting that cell number and photosynthesis are linked to the observed increase in seed weight and oil content [73]. Another growth-related TF that has been well characterized in Arabidopsis is AGAMOUS-Like15 (AGL15), which is a MADS-domain TF with the highest abundance during embryogenesis. Chromatin immunoprecipitation followed by chip hybridization (ChIP-on-chip) analysis highlighted several direct targets of AGL15, which included *LEC2*, *FUS3*, and *ABI3*. ChIP analysis revealed that HIGH-LEVEL EXPRESSION OF SUGAR INDUCIBLE GENE2 (HSI2)/VP1/ABI3-LIKE1 (VAL1) binds directly to the *AGL15* promoter through RY elements and inhibits *AGL15* expression [74]. In *hsi2-2* mutants, the expression of *AGL15* (as well as *LEC1*, *LEC2*, *ABI3*, and *FUS3*) was significantly increased compared to the wild type. These results suggest that AGL15 is also a TF that indirectly regulates oil content in Arabidopsis [74,75]. 

In the context of lipid degradation, AT-hook motif-containing nuclear localized protein 4 (AHL4) was the first TF identified in plants as likely regulating lipid degradation. AHL4 can specifically bind to the promoters of TAG lipase genes that are rich in AT motifs and to the promoters of β-oxidation-related genes, inhibiting their expression and regulating TAG degradation to affect seed germination and seedling growth progression. The seed germination rate, main root length after germination, and TAG degradation rate during seed germination and seedling establishment in Arabidopsis were all significantly higher in the *ahl4* mutant than in the wild type [76].

### 3.3. Advances in Multi-Omics Studies of Oilseed Crops

**Figure 4:**
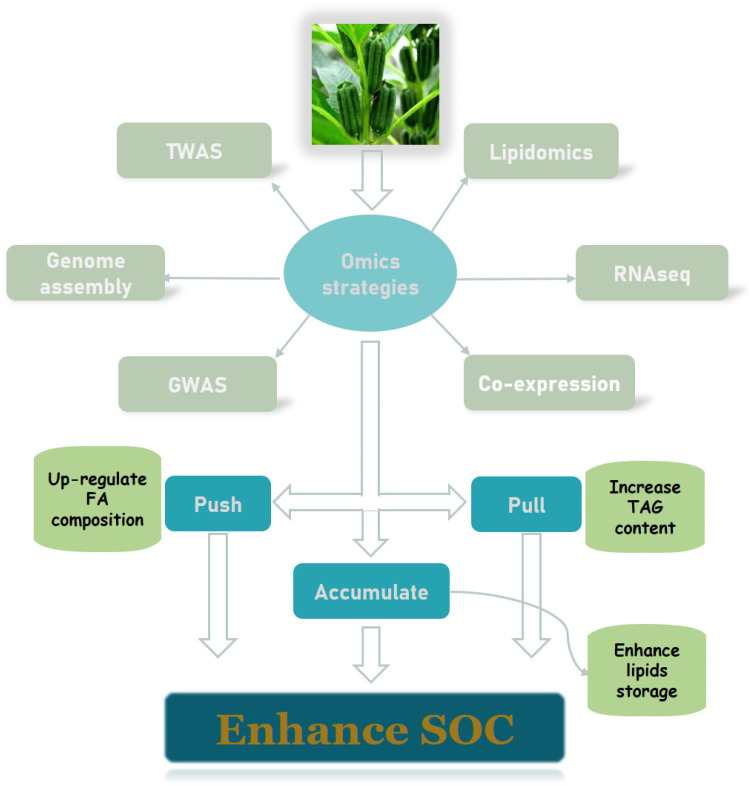
Multiple strategies to identify lipid metabolism related genes to increase SOC based on multi-omics approaches.

With the abundance of high-throughput sequencing information, the mining of large amounts of biological data is becoming increasingly popular and feasible. The availability of genomic, transcriptomic, and other multi-omics data provides a powerful avenue to unravel the detailed regulation of lipid metabolism [77,78]. From an omics perspective, the following aspects of lipid metabolism in oilseed crops are currently of interest (Figure 4).

#### 3.3.1. De Novo Genome Sequencing and Annotation

Genomic information plays a fundamental role in crop improvement programs, and large-scale population genomic analyses based on a range of genetic resources provide accurate information for identifying genomic variation underlying the selection of desirable traits [79]. In recent years, several new technologies have greatly improved existing reference genomes, including single-molecule real-time sequencing (SMRT) [80] and high-throughput chromosome conformation capture (Hi-C) technologies [81]. Continuous improvement of genome quality has greatly facilitated gene identification and deepened the understanding of crop breeding.

Taking representative oilseed genomes as an example, the first published rapeseed genome was for the winter-type European rapeseed cultivar ‘*Darmor-bzh*’, with 1097 and 1132 lipid biosynthesis genes annotated in its An and Cn subgenomes, respectively. Most of the lipid biosynthesis genes present in the genome of the rapeseed ancestor are conserved in modern-day cultivars, with the exception of 18 acyl–lipid homologs. The subgenomes An and Cn have undergone subtle structural, functional, and epigenetic cross-talk with abundant homoeologous exchanges. In addition, the selection of different rapeseed cultivars may have accelerated the loss of glucosinolate biosynthetic genes and maintained the expansion of oil biosynthesis genes [82]. Since the release of the initial rapeseed genome, reference genomes of other rapeseed varieties, including ‘*Tapidor*’, ‘*ZS11*’, ‘*Ningyou 7*’, and ‘*Da-Ae*’, have been published [83,84,85,86]. 

Using SMRT sequencing in combination with BioNano and Hi-C, the genome of peanut var. ‘*Shitouqi*’ was assembled, and the functional annotation of genes involved in seed size evolution, SOC, disease resistance, and symbiotic nitrogen fixation was performed. A total of 1944 acyl–lipid orthologs were identified in peanut, grouped into 727 gene families [87]. Chen et al. (2019) used the genome sequence of another peanut variety (cv. ‘*Fuhuasheng*’) to assess phylogenetic relationships with other legumes and oilseeds through genome and transcriptome analyses and identified more than 2500 oil-metabolism-related genes, most of which showed altered expression early in seed development and were downregulated during seed desiccation [88]. To determine the genetic basis of SOC and quality, a high-quality sunflower (*Helianthus annuus*) reference genome was assembled, allowing for the construction of a genome-scale metabolic network and the extraction of selected pathways involved in oil biosynthesis, yielding 429 genes mapped to 125 reactions corresponding to 12 pathways. A survey of the literature on sunflower oil biosynthesis showed that the network captured all 40 described genes [89]. For sesame, the genomes of several varieties have been released [90,91,92,93,94,95]; one study sequenced 705 diverse sesame varieties to reconstruct a haplotype map of the sesame genome and assembled two representative varieties de novo to identify sequence variants [92,95].

Pan-genomic analyses have aided the identification of candidate genes associated with agronomic traits by revealing many genetic variations that would not have been detected in a single genome, including large structural variants and gene fusion events [96]. In soybean, 26 representative lines from 2898 deeply sequenced varieties were selected and de novo assembled. Together with three previously reported genomes, these 26 varieties were used to construct a map-based pan-genome, followed by linking structural variants with changes in the expression of candidate genes responsible for important traits [97]. The genome of rapeseed cultivar Zhongshuang 11 (ZS11) was assembled de novo by integrating PacBio, Hi-C, and BioNano data. A comparison of the ZS11 genome to those of seven other cultivars identified millions of small variants and an average of 77.2–149.6 large presence/absence variants. More than 9.4% of the genes contained large-effect mutations or structural variations [98].

The pan-genome of cultivated sunflower was assembled to characterize its underlying genetic diversity and to quantify contributions from its wild relatives. The assembled genomes were therefore compared to those of wild species to estimate the origins of the genes present in cultivated sunflower. Analyses of allelic variation associated with resistance to downy mildew offer an example of how such introgressions from wild sunflower species have contributed to disease resistance [99]. A reference-assisted assembly approach was used to improve the draft assemblies of four sesame varieties. A comparative genomic approach was then used to analyze the five sesame genome assemblies, which were used to reconstruct the first sesame pan-genome. The results allowed for the reconstruction of the domestication history of sesame and the investigation of the gene families likely to contribute to agronomic traits [100].

#### 3.3.2. Identification of Differentially Expressed Genes

The mining of differentially expressed genes (DEGs) is a widely used strategy to study many developmental programs, including lipid metabolism [101,102,103]. Through functional enrichment analysis of DEGs between the two high-oil peanut varieties ‘*W191*’ and ‘*YH15*’ and the low-oil variety ‘*JT1*’, 57 DEGs involved in oil biosynthesis were identified, including three genes encoding ACCase, one gene encoding KASIII, one gene encoding HAD, and one gene encoding EAR. There were 11 TFs related to lipid biosynthesis. Numerous DEGs related to lipid biosynthesis were specifically observed among several peanut varieties at 25–45 days after flowering (DAF), which may represent an important stage affecting oil accumulation [104]. 

The transcriptomes of seeds and carpels at different developmental stages were determined for three sesame cultivars differing in their oil content, representing 22 datasets. A comparison of transcriptomes between sesame seeds with low and high oil content revealed more DEGs upregulated during seed development in the variety with high oil content, suggesting that seed oil plays a key role in lipid biosynthesis at later stages. Key homologous lipid genes involved in TAG biosynthesis, including those encoding GPAT, DGAT, and phospholipid:diacylglycerol acyltransferase (PDAT) at different stages of asynchronously enhanced lipid transfer protein (*LTP*) genes SIN_1019175, SIN_1019172, and SIN_1010009, are usually prominent in sesame seeds with high oil content. In addition, 23 candidate genes were identified and predicted to be beneficial for higher oil accumulation. Despite having different gene expression patterns, seeds and carpels showed a synergistic relationship during seed development [105]. Similar studies have since been performed in other species [101,106,107,108,109].

SOC accumulation rates and gene expression levels change dynamically during soybean seed development. Through the integration of DEGs and gene co-expression analysis in soybean, 124 potential genes related to oil biosynthesis were identified. Among these was *FAD2*, encoding an enzyme that catalyzes the conversion from 18:1-CoA to 18:2-PC; three other candidate genes (*GmABI3b*, *GmNFYA*, and *GmFAD2-1B*) were determined to control SOC [110]. DEG analysis was performed on seven transcriptome datasets from a pair of *Brassica rapa* accessions with different seed size, color, and oil content at different developmental stages. This analysis revealed that *BrWRI1* and *BrFUS3* consistently increase SOC and that *Bra.A03GRF5* encodes a key TF in lipid biosynthesis [111]. A comparative genome and transcriptome analysis of three crops with differences in SOC, castor bean, rapeseed, and maize (*Zea mays*) determined that over 61% of lipid- and carbohydrate-related genes were differentially expressed in castor bean and rapeseed, whereas only 20.1% (lipid-related) and 22.5% (carbohydrate-related) of the corresponding genes were regulated in maize [112].

#### 3.3.3. Construction of Oil Co-Expression Networks

Co-expression networks are another common strategy for studying the transcriptional regulation of lipid metabolism based on multi-omics data, among which weighted gene co-expression network analysis (WGCNA) is the most widely used method [113,114]. Co-expression gene networks have been widely applied in several species [110,111,115,116].

A multi-level approach was implemented in oil palm combining WGCNA, quantification of allele-specific gene expression, and joint multivariate analysis of the transcriptome and lipidome to an interspecific backcross population between the African oil palm and the American oil palm, which have contrasting oil contents and FA compositions. The resulting gene co-expression network revealed a close transcriptional coordination of FAS in plastid with sugar sensing, plastid glycolysis, transient starch storage, and carbon recovery pathways [117]. An integrated co-expression network analysis among Arabidopsis, rapeseed, soybean, and sesame revealed that three soybean genes (Glyma.13G148600, Glyma.13G207900, and Glyma.12G122900) were co-expressed with soybean phosphoenolpyruvate carboxylase 1 (*GmPEPC1*), while *BnPEPC1* transcript levels were repressed by the microRNA bna-miR169, which correlated with SOC. In addition, two rapeseed-specific genes encoding heterologous ACCase subunits β-CT (BnaC05g37990D) and biotin carboxyl carrier protein 1 (BCCP1) (BnaA03g06000D) were identified, which may affect oil biosynthesis. Notably, these genes are thought to negatively regulate oil biosynthesis [115]. 

The genotype data of 398 soybean recombinant inbred lines were combined with measurements for 249 metabolites by gas chromatography–time-of-flight mass spectrometry (GC-TOF MS), resulting in 142 oil biosynthesis genes and 326 lipid metabolism genes extracted from the assembled multi-dimensional genetic network. Using information from DEGs specifically expressed in seeds and genes homologous to Arabidopsis lipid-related genes, six modules related to lipid metabolism were clustered [118]. A WGCNA was performed using 24 RNA-seq libraries collected from eight post-pollination growth periods of walnut (*Juglans regia*) seeds, and 6890 DEGs were grouped into six modules. These genes were ranked, and the top 19 were selected, among which *ACP*, *VESICLE-ASSOCIATED MEMBRANE PROTEIN 727* (*VAMP727*), and *INDETERMINATE DOMAIN 14* (*IDD14*) were co-expressed with *WRI1* [113].

#### 3.3.4. Genome-Wide Association Studies to Map Oil Content–Related Loci

Genome-wide association studies (GWASs) analyze the association between traits of interest and markers or candidate genes based on linkage disequilibrium between loci within a population, providing a new research strategy for mining genomic regions of importance [119]. A GWAS-based analysis using whole-genome resequencing of 418 rapeseed varieties identified 628 candidate genes related to 56 important agronomic traits, including SOC. In addition, nonsynonymous mutations were uncovered in plausible candidate genes for these agronomic traits that showed significant differences in their allele frequencies across rapeseed improvement, including the ribosome recycling factor (*BnRRF*) gene for seed weight. The genetic network underlying 17 traits including seeds per pod and FA components was further characterized by the linkage disequilibrium of associated loci with phenotypic correlation [120].

A resequencing effort with 302 soybean accessions facilitated a GWAS that revealed associations between 10 selected regions and nine domestication traits and identified 13 new loci associated with agronomic traits, including SOC. When integrated with previous QTL information, the GWAS established that, of the 230 detected selective sweeps, 96 correlated with previously reported oil-related QTLs. Importantly, 21 of the mapping intervals for these QTLs contained FA biosynthesis genes. In addition, some traits and loci appeared to be associated with geographical regions, suggesting that soybean populations are geographically structured [121]. Comparative genomic and transcriptomic analyses in peanut revealed 147 common gene clusters, and a GWAS identified 48 important insertion/deletion (InDel) markers associated with SOC in five environments with the best linear unbiased prediction value. In addition, one InDel cluster mapped to chromosome A03 and was associated with SOC variation in three environments; the related candidate genes presented variable alleles that differed in their oil content in an independent population [122]. 

By exploiting the natural variation in 705 cultivated sesame varieties and performing a large-scale GWAS on 56 agronomic traits, 549 key genomic loci underlying oil content, nutritional quality, and oilseed yield of sesame were systematically identified. Forty-six candidate genes were identified by integrating functional genomic information. Several of the candidate genes for SOC encode enzymes involved in oil metabolism, and two major genes associated with lignification and black pigmentation in the seed coat were also associated with large variations in oil content [93]. Previous studies have shown that oleic acid content is a polygenic trait, although no stable QTL has been reported outside *FAD2* [123,124,125]. The genotypes of 375 rapeseed accessions with low erucic acid content were used for a GWAS, which was combined with a high-density genetic map for a 150 double haploid population to identify a new QTL on chromosome A9. This new locus explained 11.25%, 5.7%, and 6.3% of the phenotypic variation over three consecutive growing seasons and increased the oleic acid content by about 3–5%. In addition, three potential candidate genes related to FA biosynthesis were found and verified the FAs in a homologous gene mutant of Arabidopsis by fine-mapping and gene expression analysis [126]. 

Accurately interpreting and selecting candidate single-nucleotide polymorphisms that are highly correlated with complex traits has become a bottleneck in understanding how genes affect traits in detail [127,128]. Recent studies have shown that co-expression networks can provide a guide for prioritizing candidate genes from GWAS regions, including the detection of quantitative trait genes (QTGs) and genes related to lipid metabolism. The computational framework Camoco (co-analysis of molecular components), which integrates GWAS-identified loci with functional information from gene co-expression networks, detected the accumulation of 17 different elements in maize seeds from a large-scale GWAS dataset along with two candidate genes validated by mutants [129]. QTGs in rapeseed were delineated by coordinating the expression of genes located within QTLs and known orthologs associated with Arabidopsis traits, and a 21-module co-expression network of acyl–lipid metabolism was established, consisting of 270 known acyl-lipid genes and 3503 new genes. The core module contained 76 known genes involved in FA and triacylglycerol biosynthesis [130]. Based on GWAS and co-expression analysis, six haplotype blocks associated with oleic acid (C18:1) were identified in rapeseed, located on chromosomes A02, A07, A08, C01, C02, and C03. In addition, three genes (*BnmtACP2-A02*, *BnABCI13-A02*, and *BnECI1-A02*) on chromosome A02 and two genes (*BnFAD8-C02* and *BnSDP1-C02*) on chromosome C02 were closely associated with phenotypic variation that may be regulated by oleic acid accumulation [116].

#### 3.3.5. Lipidomics for Oil Structure and Quality Identification

Lipidomics is now considered a systematic research pattern based on high-throughput liquid chromatography–tandem mass spectrometry (LC-MS/MS) analysis, which can be used to determine the composition and changes in the abundance of the lipidome in organisms [131]. Lipidomic analysis of developing rapeseed seeds showed that initial lipid deposition begins in the aleurone layer and endosperm. As the seed develops, lipid accumulation in the embryo increases significantly and the distribution of molecular species changes significantly from early (20 DAF) to intermediate (27 DAF) stages of lipid accumulation [132]. In another study, the spatial distribution of two major lipid classes, TAG and PC, was investigated using matrix-assisted laser desorption/ionization-mass spectrometry imaging (MALDI-MSI). Significant differences in lipid composition were observed between different tissue types within rapeseed seeds: Palmitic acid was enriched in the hypocotyl, whereas oleic acid and linoleic acid accumulated in the testa/aleurone layer. In addition, the lipid compositions of the inner and outer cotyledons differed significantly [133]. To analyze the lipid composition of tea tree (*Camellia sinensis*) seed oil and to reveal the functional properties of different lipid components, ultra-performance liquid chromatography–tandem mass spectrometry (UPLC-MS/MS)-based lipidomics was used to characterize the lipid profile; this showed that seed oil has a more diverse range of glycerides in tea tree seeds, with a relatively higher content of glycerol diesters, compared to other plant oils [134]. 

To investigate the effect of host carbon supply on nodulation and nitrogen fixation in the interaction between symbiotic rhizobia and legumes, researchers performed lipidomic and transcriptomic analyses on soybean roots and nodules at six different developmental stages. The lipidomic data revealed adaptive changes in lipid metabolism during nodule formation that affect rhizobial proliferation, growth, and differentiation and demonstrated that the biosynthesis and transport of FAs, monoacylglycerol (2-MAG), and membrane lipids may be a prerequisite for symbiotic success [135]. The advantage of lipidomic analysis lies in its ability to accurately quantify and identify lipids; despite certain advances in food component characterization and quality identification, lipidomics as a new emerging discipline started relatively late compared to other omics fields [136,137,138,139]. Currently, the successful application of lipidomics in the characterization of plant oils and fat composition is still limited, and this line of research still faces many challenges [140]. In addition, by combining lipidomics with other omics information, we hope to identify the types and levels of lipids in oilseed crops at different developmental stages, thereby studying the pattern of lipid accumulation and discovering ways to improve oil content.

## 4. Conclusions and Future Prospects

With the rapid development of multi-omics and molecular biology techniques, research on the regulatory mechanism of oil biosynthesis in oilseed crops has accelerated. Three main strategies have been proposed to enhance SOC, namely “push” (upregulate FA content), “pull” (promote TAG assembly), and “accumulate” (enhance TAG storage or inhibit TAG degradation) [57,141]. At present, the basic pathways regulating lipid biosynthesis in plants have been elucidated, and many essential genes and regulatory factors have been identified, which could provide positive guidance for the molecular breeding of oilseed crops. It should be noted, however, that the regulation of oil biosynthesis is a complex biological program and a quantitative trait controlled by multiple genes. The effect of simply changing the expression level of one or a few genes on oil content and composition may be relatively limited. Although QTL mapping and GWASs based on large populations have been widely applied in rapeseed, soybean, and other oilseed crops, more sequencing data together with more diversified analytical strategies are needed for genome assembly, investigation of linkage disequilibrium, and functional verification.

More attention should also be paid to the integration of gene sets related to lipid metabolism in oilseed crops. Most of the current functional studies on synthetic regulatory genes rely on the reported Arabidopsis homologous dataset, which was published in 2010 and has not been updated since [44]. In addition, since Arabidopsis (although related to Brassica oilseed crops) is not a typical oil crop, direct comparison based on this dataset is likely to result in loss of information. Literature mining and integration of the current database may well complement the homologous gene set, which could serve as an important reference for the study of plant oil metabolism. The search for genes unique to different oilseed crops is a very active area of research. While the biosynthetic pathways show similarities, the resulting lipid products vary considerably among oilseed crops, highlighting the critical role of species-specific genes. The identification of such genes can be facilitated by the concept of de novo genome assembly and annotation, enabling the discovery of novel species-specific genes. By using a well-constructed collection of homologous genes associated with lipid metabolism in a specific species, the expression levels of these genes can be evaluated using transcriptome datasets. Co-expression networks can be used to assess the strong associations between these homologous genes and the diverse end products observed in these oilseed crops. In addition, with sufficient knowledge of the chemical formulas of these end products and precursor structures, the species-specific candidates may be predicted ab initio by transforming them into a SMILES (Simplified Molecular Input Line Entry System)-like format and combining them with computer-assisted search planning (CASP) strategies, including deep learning.
